# Supplementary material for: Using decoys and camera traps to estimate depredation rates and neonate survival
Source: PLoS One. 2023 Oct 24;18(10):e0293328. doi: 10.1371/journal.pone.0293328 (PMC10597525; doi:10.1371/journal.pone.0293328)
Supplement: S1 Table — (DOCX) [file pone.0293328.s001.docx]

**S1 Table.** **Cost analysis comparing a typical live-capture neonate project design vs. a camera trap survey paired with decoys and scent lures. Numbers are adjusted to obtain data on 100 neonates’ survivorship. Team member wage assumed to be $10.00/hr. Camera survey methods can be used twice in a neonate birthing period. We assumed a 1:1 return on investment for VIT:fawns captured.**

|  | **Live-Capture Fawn Method** | | | | | **Camera Trap and Decoy Method** | | | | |
| --- | --- | --- | --- | --- | --- | --- | --- | --- | --- | --- |
| ***Supplies*** | **Item** | **Quantity** | **Individual Cost $** | **Total Cost ($)** | **Reusable?** | **Item** | **Quantity** | **Individual Cost $** | **Total Cost ($)** | **Reusable?** |
|  | ***Doe Collar*** | 100 | 182 | 18,200 | No | **Camera Traps** | 100 | 200 | 20,000 | Yes |
|  | ***Expandable Collar*** | 100 | 220 | 22,000 | No | **Fawn Decoys** | 50 | 35 | 1,750 | Yes |
|  | ***Vaginal-Implant Transmitter (VIT)*** | 100 | 268 | 26,800 | No | **8x5in Carpet Square** | 50 | 0.50 | 25 | Yes |
|  | ***Radio Receiver*** | 2 | 1,000 | 2,000 | Yes | **Fawn Urine Scent Lure** | 6 | 8 | 48 | No |
|  | ***Radio Antenna*** | 2 | 150 | 300 | Yes | **SD Cards** | 100 | 6 | 600 | Yes |
|  | ***Dart Gun*** | 1 | 2,000 | 2,000 | Yes | **Batteries** | 800 | 1.25 per battery | 1,000 | If rechargeable, yes |
|  | ***Darts*** | 30 pkgs (5/pkg) | 18 | 540 | No | **Python Locks** | 100 | 12 | 1,200 | Yes |
|  | **CO2 Cartridge** | 24 | 100 | 2,400 | No |  |  |  |  |  |

|  | **Tranquilizer Drugs such as BAM** | 10 vials | | 265 | | 2,650 | | No |  |  | | |  | |  |
| --- | --- | --- | --- | --- | --- | --- | --- | --- | --- | --- | --- | --- | --- | --- | --- |
|  | **Bait** | 40  (40kg of corn/bag) | | 7 | | 280 | | No |  |  | | |  | |  |
|  | **Supplies Approximate Cost: $77,170** | | | | | | | | **Supplies Approximate Cost: $24,623** | | | | | | |
| ***Survey Effort*** | **Action Item** | | **Total Hours** | | **Number of Personnel** | | **Total Cost $ (assuming $10/hr)** | | **Action Item** | | **Total Hours** | **Number of Personnel** | | **Total Cost $ (assuming $10/hr)** | |
|  | **Capture / Collar Pregnant Does (Spring)** | | 1,060 | | 2 | | 21,200 | | **Camera Set Up** | | 600 | 1 | | 6,000 | |
|  | **Monitor VIT Signals** | | 1,680 | | 1 | | 16,800 | | **Decoy Set Up** | | 384 | 1 | | 3,840 | |
|  | **Initial Fawn Recovery** | | 100 | | 1 | | 1,000 | | **Photo Processing** | | 360 | 1 | | 3,600 | |
|  | **Final Fawn Recovery** | | 100 | | 1 | | 1,000 | |  | |  |  | |  | |
|  | **Office Data Processing** | | 50 | | 1 | | 500 | |  | |  |  | |  | |
|  | **Survey Effort Approximate Cost: 40,500** | | | | | | | | **Survey Effort Approximate Cost: 13,440** | | | | | | |
|  | **Project Total Cost: $117,670** | | | | | | | | **Project Total Cost: $38,063** | | | | | | |
