# Supplementary material for: Using decoys and camera traps to estimate depredation rates and neonate survival
Source: PLoS One. 2023 Oct 24;18(10):e0293328. doi: 10.1371/journal.pone.0293328 (PMC10597525; doi:10.1371/journal.pone.0293328)
Supplement: S2 Table — (DOCX) [file pone.0293328.s002.docx]

| State of Study | Probability of Survival (%) | *# of neonates monitored* | # of neonates censored due to method related issues | % Unknown Mortality | Rates of Depredation (%) | | | | | | | Source |
| --- | --- | --- | --- | --- | --- | --- | --- | --- | --- | --- | --- | --- |
|  |  |  |  |  | Coyote | Bobcat | Black Bear | Black Vulture | Gray Fox | Gray Wolf | Unknown-Uncertain Predator |  |
| NC | 31 | 122 | 0 | - | 17 | 7 | 44 | 5 |  | - | 0 | This Study |
| LA | 27 ^e^ | 70 | 0 | 2 | 18 | 22 | 33 | 0 | 0 | - | 16 | Shuman et al. 2017 |
| NC | 14 ^d^ | 65 | 1 | 6 | 55 | 9 | - | 0 | 0 | - | 0 | Chitwood et al. 2015 |
| GA | 29 | 47 | 4 | 19 | 54 | 10 | - | 0 | 0 | - | 14 | Nelson et al. 2015 |
| SC | 22 ^d^ | 91 | 0 | 0 | 37 | 7 | . | 0 | 0 | - | 26 | Kilgo et al. 2012 |
| MI | 36 ^a^ | 100 | 22 | 3 | 11 | 6 | 11 | 0 | 0 | 4 | 23 | Kautz et al. 2019 |
| AL | 33 ^a^ | 36 | 0 | 6 | 100 | 0 | - | 0 | 0 | - | 0 | Saalfeld and Ditchkoff 2007 |
| SD | 75 ^b^ | 81 | 0 | 0 | 83.3 | 0 | - | 0 | 0 | - | 17 | Grovenburg et al. 2012 |
| PA | 53 ^a^ | 218 | 21 | 2 | 37 | 6 | 33 | 0 | 0 | - | 24 | Vreeland et al. 2004 |
| WI | 45 ^b^ | 139 | 1 | 7 | 60 | 7 | 7 | 0 | 0 | - | 20 | Warbinton et al. 2017 |
| WI | 65 ^b^ | 89 |  | 12 | 14 | 23 | 26 | 0 | 0 | - | 37 |  |
| SC | 68 | 210 | 2 | 32.4 | 48 | 24 | . | 0 | 0 | - | 28 | McCoy et al. 2013 |

**S2 Table.** **White-tailed deer neonate survival, predator species-specific rates of depredation, and rate of uncertainty in mortality for studies of fawn survival across North America. Numbers used to represent this study are based on the first predator event per site only. - = species not present at the study site. % of predation was calculated from the total number of mortality events caused by a predator divided by the total number of dead neonates.**

^a^ Survival to 180 day, ^b^ Survival to 110 days, ^c^ Survival to 140 days, ^d^ Survival to 112 days, ^e^ Survival to 84 days, ^f^ Survival to 154 days

References:

Shuman RM, Cherry MJ, Simoneaux TN, Dutoit EA, Kilgo JC, Chamberlain MJ, Miller KV. Survival of white-tailed deer neonates in Louisiana. Journal of Wildlife Management. 2017;81(5):834–845.

Chitwood MC, Lashley MA, Kilgo JC, Pollock KH, Moorman CE, DePerno CS. Do biological and bedsite characteristics inﬂuence survival of neonatal white‐tailed deer? PloS ONE. 2015;10(3):e0119070.

Nelson MA, Cherry MJ, Howze MB, Warren RJ, Conner LC. Coyote and Bobcat Predation on White-tailed Deer Neonates in a Longleaf Pine Ecosystem in Southwestern Georgia. Journal of the Southeastern Association of Fish and Wildlife Agencies. 2015;2:208-213.

Kilgo JC, Ray HS, Vukovich M, Goode MJ, Ruth C. Predation by coyotes on white-tailed deer neonates in South Carolina. Journal of Wildlife Management. 2012;76:1420-1430.

Kautz TM, Belant JL, Beyer DE, Strickland BK, Petroelje TR, Sollmann R. Predator densities and white‐tailed deer neonate survival. Journal of Wildlife Management. 2019;83(5):1261–1270.

Saalfeld ST, Ditchkoff SS. Survival of neonatal white-tailed deer in an exurban population. Journal of Wildlife Management. 2007;71:940–944.

Grovernburg TW, Jenks JA, Jacques CN, Klaver RW, Swanson CC. Aggressive defensive behavior by free-ranging white-tailed deer. Journal of Mammalogy. 2009;90:1218-1223.

McCoy JC, Ditchkoff SS, Raglin JB, Collier BA, Ruth C. Factors Influencing Survival of White-tailed Deer Neonates in Coastal South Carolina. Journal of Fish and Wildlife Management. 2013;4(2):280–289.

Vreeland JK, Diefenbach DR, Wallingford BD. Survival rates, mortality causes, and habitats of Pennsylvania white-tailed deer neonates. Wildlife Society Bulletin. 2004;32:542-553.

Warbington CH, V. Deelen TR, Norton AS, Stenglein JL, Storm DJ, Martin KJ. Cause-specific neonatal mortality of white-tailed deer in Wisconsin, USA. Journal of Wildlife Management. 2017;81(5):824–833.
