# Supplementary material for: Using decoys and camera traps to estimate depredation rates and neonate survival
Source: PLoS One. 2023 Oct 24;18(10):e0293328. doi: 10.1371/journal.pone.0293328 (PMC10597525; doi:10.1371/journal.pone.0293328)
Supplement: Attachment — Submitted filename: PONE-D-23-13153_review.pdf [file pone.0293328.s004.pdf]

Review for:

Using decoys and camera traps to estimate depredation rates and neonate survival

In this paper the authors were interested in evaluating a new method to estimate neonate survival using camera traps. The paper has a logical flow to it, which makes it easier to understand, though some of the paragraphs could use some restructuring to improve flow a little more (e.g., the introduction). I thought that the suggested method was interesting, and the authors could even go a bit further to sell the fact that this technique is non-invasive (brought up a few times at the start but really only referenced quickly again in the discussion). However, I did have some concerns with the analysis. The biggest issue is how the analysis was described in the methods (see below), but also I wonder why the authors did not account for or at least explain why they did not account for uncertainty in predator presence on the landscape and also what the relative predator activity metric represents ecologically. More specifically:

1. The reporting of the methods leaves a lot of uncertainty with respect to how the models were fit, and what data was used. For example, it is still unclear to me if there was a pre-decoy and post decoy model fitted to the data (i.e., separate models for each species) or if the authors included a 'decoy' dummy variable. This is especially important to cover because the authors try to make inference about a decoy effect, or rather that there is none, and I cannot determine how their model actually did this based on the covariates they said they included. This makes it so that a reader would not likely be able to recreate the authors analysis from the information presented, which is a problem.

2. It is possible to account for uncertainty in predator presence on the landscape while fitting your survival models. There are a few way you could do this. First, if you are interested in predator specific effects, you could just fit an occupancy model with a time to detection observation model. This is something covered in depth (model and code) by Kéry & Royle (2015) and also something I used in Fidino et al. (2020, with data and code here). Conversely, you could fit standard occupancy models and then link that with the survival model. For a frequentist analysis, this could be done by using a bootstrap method and estimating predator occupancy at each site (leaving you a distribution of parameters for that term). For a Bayesian analysis you could just link the models together and fit the models at the same time (i.e., link a multi-species occupancy model with a survival model). This could, for example, allow you to estimate 1) the effect of multiple predators being present by summing across the predator incidence matrix or 2) the effect of individual predators with dummy variable. Finally, with a Bayesian analysis you could even use a Poisson distribution for the observation model, treating the number of times a species was detected (e.g., photos) as the response variable. By summing that across your predators you could have a 'detection corrected' relative abundance metric. And then from there you could link your a Bayesian survival model to this third type of model. Basically, because there are multiple avenues available to the authors, it strikes me as odd to assume no observational error occurs during data collection, which is what they are currently doing. I'm not encouraging a full reanalysis here, but some justification of their baseline assumptions they are making would help. For example, one relatively 'simple' way to justify their assumption is to fit some

occupancy models with their predator data to estimate predator detection probabilities. The authors could then calculate the probability each predator is detected at least once with the number of survey days you have available (1 - (1 - p)^j where j is the number of days sampled). This information could then be shared so that the reader has an idea about how much each species may be contributing to the predator relative abundance metric, and if there are some species who are regularly present or absent from that covariate.

Fidino, M., Barnas, G. R., Lehrer, E. W., Murray, M. H., & Magle, S. B. (2020). Effect of lure on detecting mammals with camera traps. Wildlife Society Bulletin, 44(3), 543-552.

Kéry,M.,and J.A.Royle.2015. Applied hierarchical modeling in ecology: analysis of distribution, abundance and species richness in R and BUGS. AcademicPress,London,England,UnitedKingdom.

3. The predator relative abundance metric comes out as important in all of your top models. However, I am having a hard time trying to interpret what that would actually mean with respect to neonate survival. To me, it would seem as if each predator would vary in how successful they are with finding your decoy, and this metric assumes that predators are essentially exchangeable. Some more justification for this metric would help, and also some guidelines for how this should be interpreted ecologically. For example, the authors bring up how traditional methods make it difficult to determine the predator, while camera traps do make it possible. To me, that seems to indicate an interest in understanding predator specific effects, which this predator relative abundance metric does not capture.

I think that the authors can get passed a lot of these issues with some additional explanation / logic, which would not require a full reanalysis. Certainly, if the authors want to reanalyze their data to account for such issues they could, but at a minimum they would need to provide some caveats about the current statistical approach (and even indicate that these caveats could be solved with different styles of analysis).

If the authors have any specific questions for me about this review I can be reached at mfidino@lpzoo.org. I have section specific comments below, I hope the authors find them helpful.

Cheers, Mason Fidino

Abstract

Line by line comments

line 26: To split up the commas a bit it would be good to use em dashes for `individuals less than four weeks old`. 100% my own writing style creeping in here, so feel free to ignore!

Line 27: `population dynamics of a population` is redundant. Just say "...can influence ungulate population dynamics" or something like that.

Line 39: You can definitely report probabilities at percentages like this, but when you do that it gets a little weird when you need to report uncertainty (e.g., 95% confidence intervals). For example, 31% (95% CI = 22%,35%) looks off given the multiple percentages. Instead, 0.31 (95% CI = 0.22, 0.35) looks much cleaner (and even saves space).

Introduction

Top-level thoughts

1. The first paragraph could probably get split into two. The first half is about neonate survival in ungulates while the second half is about how researchers estimate neonate survival. As such, the topic sentence does not really line up with half the content of this paragraph. You could get around this by adding a closing sentence after line 56 and then have the 'consequently' sentence there be the topic sentence of a new second paragraph. Further, what is currently paragraph 2 in this draft has a mix of redundant information and then more specificity than what is currently the second half of paragraph 1. If you split this paragraph you can fold in a lot of info from the current second paragraph / remove redundancies.

2. Making predictions with the 'average' model in this case is not good because the nest survival model does not use the identity link (at least in terms of calculating average parameters and then using those for predictions). This is covered at length by `Cade (2015)`. However, `Banner and Higgs (2017)` have some pointers on how this may actually be done. Chances are, if that authors used the standard approaches available, they first generated average parameter values and then used them for prediction. If that is the case, that is not the correct thing to do with this model (I believe you can model average predictions instead). Furthermore, the notion that you can get an idea about the 'overall effect' of a given parameter via model averaging is not really correct, and this error is unfortunately quite pervasive in our field. You might instead consider that you have quite a few uninformative parameters in your competing models (e.g., if they are nested subsets of one another), and if that is the case, you may just need to work off a single model (see Arnold 2010).

Arnold, T. W. (2010). Uninformative parameters and model selection using Akaike's Information Criterion. The Journal of Wildlife Management, 74(6), 1175-1178.

Banner, K. M., & Higgs, M. D. (2017). Considerations for assessing model averaging of regression coefficients. Ecological Applications, 27(1), 78-93.

Cade, B. S. (2015). Model averaging and muddled multimodel inferences. Ecology, 96(9), 2370-2382.

Line by line comments

line 52: ...can have **a** greater influence...

Line 53-54: This sentence comes a bit out of left field. If variation in neonate survival is important, how does having reliable measurements of this variation inform adaptive management? Is it to control the variation? To increase survival? A little more clarity here would help to make sure the reader follows your logic. I don't disagree with your statement, but right now you are forcing the reader to fill in some logical gaps in order to agree with this.

Line 56: You can drop the 'also'

Line 63 - 64: This sentence is redundant with info from the previous paragraph.

Line 67: Change 'An alternative method' to 'Alternative methods' as you list multiple methods here.

Line 72: Missing a closing sentence on this paragraph.

Line 82 - 83: `...a cheaper and less intrusive method...`. Right now there is uncertainty if 'less' should be applied to the word 'intrusive.'

Line 95 - 102: Since the topic here is predation it could also make sense to cite some paper that used decoy eggs for nest predation.

Line 104: considering or assuming? You likely also need to defend this assumption / provide some logic behind it. Two come to mind to be: as 'hider' species, if a predator gets within that distance they are 1) not likely to miss the prey and 2) the prey lacks a strategy to escape the predator at that distance.

Line 110: You've been past tense with this paragraph so it would read better to say 'we predicted that depredation rates...' Also, why did you make this prediction?

Methods

Top-level thoughts

1. Why make predictions for only three predators when more are present?

2. If the goal was to limit an individual predator visiting multiple camera trapping locations the clustered design only really achieves that at the 'among-cluster' scale for many of these species (i.e., 250 m is still quite close for larger ranging species like black bear or coyote). This may cause some small amount of non-independence among camera trapping locations, which at a minimum should be acknowledged here.

3. Figure 2 was so small in the review I could not see it, but I'll take your word that the image gets the point across.

4. Your analysis does not account for the clustered nature of your study design. A cluster-based random effect would do this, but is unfortunately not available in `RMark`.

5. One thing that is not made clear in the methods is how the effect of neonate was quantified. I did not see any explanation about whether the first two weeks of data was used in the analysis or if that was simply done for acclimation. To me, it would seem like you could estimate weekly time to detection with all the data and include some binary variable to indicate a predation event (though there is some complication about what you would consider a predation event when

the neonate decoy is not present). Regardless, some extra information here is welcome (note: looking at the results it seems like models were fit to each period seperately, not seeing where this is brought up in the methods though I could have missed it).

6. Using a single person for camera trap surveys makes the assumption that this is safe to do so, which is not always the case and may not consider the risks that marginalized communities face in our field (Rudzki et al. 2022 and references therein). For example, currently the camera trap method only requires 45% of the number of survey hours as the other method (line 350). Does that mean if two people were required it would be 90%? It could help to recognize that multiple people may be necessary depending on how safe it is to conduct fieldwork.


```
Rudzki, E. N., Kuebbing, S. E., Clark, D. R., Gharaibeh, B., Janecka, M. J., Kramp,
R., ... & Richards-Zawacki, C. L. (2022). A guide for developing a field research
safety manual that explicitly considers risks for marginalized identities in the
sciences. Methods in Ecology and Evolution, 13(11), 2318-2330.
```


Line by line comments

Line 133 - 134: How was the proportion of conifer / hardwood overstory quantified?

Line 165: You say 1 dm here than 10-cm everywhere else. Use one or the other (though they are equivalent).

Line 184 - 190: How did you measure 2.5 meters from the neonate in the videos?

Line 201 - 211: How was this measured (was not covered in the methods so far).

Line 232 - 233: Why fit the models seperately for inside and outside black bear range? Could you have included a dummy variable there for black bear (plus interactions with other covariates) instead? You'd have the same number of parameters to estimate, but you'd be sharing information for other species which could leave to more precise estimates.

Line 233 - 235: So are we assuming that all possible combinations are biologically reasonable?

Results

Top-level thoughts

1. What was the naive occupancy of these different predators across the sampled areas? Right now your model assumes that, aside from the black bear, these species are available to prey on your decoy (or more specifically that they are present in each location).

Line by line comments

Line 271 & 272: The reported detection rates are a little confusing because the second ones (0.035 & 0.004) lack the `/day` component.

Line 272 - 276: This was not covered in the methods, but did you fit the models separately (a pre-decoy model and a post decoy model)? If you did, you did not

actually make any statistical comparison about a decoy effect here, meaning this sentence is not correct. Second, if you did quantify this effect, failing to detect an effect does not mean that there is no effect (i.e., you should not say there was no change). Instead, you failed to detect a difference. But again, if these were separate models you did not quantify a difference.

Line 280 - 290: How were all these depredation probabilities calculated? Given the lack of uncertainty in the estimates, it seems like these are raw summaries of the data? However, if that is the case, these estimates would be biased as they do not account for the fact that your data are right-censored (which is what your nest survival model accounts for). These depredation probabilities can be made from your model, and as such could also come with an uncertainty estimate. For example, you could calculate the cumulative probability that a neonate gets predated over the course of your study for each species (or even the cumulative probability a neonate gets predated by any species, perhaps inside and outside of black bear range).

Discussion

Top-level thoughts

1. One thing that is not really brought up is that there is no doubt spatial variation in deer across the landscape. How are camera trapping sites selected relative to this? Are you only sampling in areas where deer are known to occupy with a high likelihood?
2. The concluding paragraph lists some future directions but does not really bring us back to the contribution provided by this research. I'd circle back at the end to remind the reader about the key takeaways here / why this research helps move this field forward.
3. Do the authors have any tips for people who are interested in using this method?

Line by line comments

Line 369 - 370: How is the camera-decoy approach not perfect (i.e., give an example or two to the reader about this).

Line 387 - 391: Again, I'm not sure that the authors specifically evaluated this effect given that they fit separate models to different subsets of the data, which means that they did not statistically evaluate / quantify a difference. Similarly, the authors calculated species-specific depredation rates from the raw data when it could have been estimated in the model. As such, the authors could make descriptive comparisons about their sample but it is a bit more difficult to assume that these value are representative of the larger population (i.e., using inferential statistics to get said estimates). As this paper is more methodological, some care should be taken here to not oversell what this technique can provide. For example, would it be necessary to get a much larger sample size to estimate predator specific effects? If so, what do the authors suggest?

Tables & figures

ALL TABLES: Numeric columns should be right aligned and text columns should be left aligned. Right now it looks like the numeric columns are center aligned.

Table 3 - 5. Saying S = survival probability is a little odd. Looking at the models it appears to be a function in that parentheses are used. Probabilities are not functions, though they may be a function of different variables. Maybe instead of survival probability you sould just remove the 'S()' part and say that this is the linear predictor for the survival model? That would be more explicit about what that part of the table represents.

Table 3-5. defining delta AICc as delta AICc is not super helpful for people who may be a little unsure of this metric. Instead you could define it as the difference in AICc from the AICc score of the model with the relative best fit? You've also not said what the weight column is in these tables.

Table 6. You could separate the 95% CI's with a comma instead of |.

Figure 2. `ggplot2` defaults to grey axis text, which is not ideal. I don't use that package, but I think something like `theme_bw()` should update those so that they are black instead.

Figure 5. Please include 95% CI for these lines. Tick marks on the x and y axis would also be helpful.
